# Editors’ Book Table

**Published:** 1872-12

**Authors:** 


### Editors' Book Table.

[NOTE. — All works reviewed in the columns of the CHICAGO MEDICAL JOURNAL may be found in the extensive stock of W. B. KEEN, COOKE & Co., whose catalogue of Medical Books will be sent to any address upon request.]

BOOKS RECEIVED.

*The Medical Register and Directory.* Chicago, 1872–3. Edited and published by T. DAVIS FITCH, M.D., and NORMAN BRIDGE, M.D. Revised by the Presidents of the regular Medical Colleges and Societies of Chicago, and of the Illinois State Medical Society.

The prospectus of this little work having been already noticed in this journal, it only remains for us to say how far the work has verified the promises of its projectors.

In this regard we are glad to be able to congratulate the editors upon having violated the usual rule applying to promises, for they have done all that they promised, and more too, for they have adopted some suggestions thrown out by the JOURNAL, in its remarks upon their prospectus, which have added to the value and popularity of the work.

We trust that the work will be—is—a permanent institution, and will in a little while become a necessity to every physician in the State.

It is gratifying to see that the editors have had the moral courage to follow the example set by the Relief Committee last winter, in applying the amputating knife liberally, and lopping off excrescences and parasites from the professional body, which will receive renewed health and increased vigor from the operation. They have done well! and we trust they will sharpen up their knives and reheat their cauteries, for another application, before the issue of the next edition.

The work itself might be somewhat reduced in bulk, and advantageously too. The omission of the History and Code of Ethics of the American Medical Association, would be no loss to the work, and a decided gain to its readers, as we imagine very few are now interested in that ingenious method of cheap advertising, which has long since outlived its usefulness even for that purpose.

The code of ethics of the medical profession is simply a verbal amplification and concrete application of an older and more venerable code, promulgated by a higher authority: "Do unto others as you would they should do unto you." He who appreciates this, can be trusted without the code—he who does not, cannot be trusted under its most rigid application. We hope to see in the next edition of this work, an alphabetical list of physicians, with the places of their graduation, and dates of their diplomas affixed. H.

*Hand Book of Compound Medicines, or Prescribers' and Dispensers' Vade Mecum.* By ARNOLD J. COOLEY. London. Philadelphia: J. B. Lippincott & Co. 1873. Pp. 219.

A small volume of considerable information, rather intended for junior practitioners. It is an epitome of the prescriptions of the leading hospitals and physicians and surgeons of London. English doctors use more medicines than Americans, and the formulæ contained in this volume indicate drugs that will kill or cure. Very many of the prescriptions produced would not be used by the average American graduate, because he knows of remedies that would accomplish his purpose in a pleasanter way. No attention is paid—so it would seem—to freeing pills and potions from their native nauseousness and abominableness. Sugar coating for pills is alluded to as "The *Sugar Coated Pills of Quacks.*" Every American druggist can sugar-coat pills in a few moments, and the quackishness of the resort has never before come to our notice.

*Very many* good formulæ are contained in this small volume, and with the good ones are many that are simply atrocious. Vide p. 9. "Anti-bilious Pill." Here are the ingredients without the quantities: "Aloes—Colocynth—Rhubarb—Myrrh—Scammony—Ipecacuanha—Cardamom Seeds—Medicinal Soft Soap—Oil of Juniper, and Treacle"! And this is offered as an anti-bilious pill.

On the whole, the book may be said to contain prescriptions of English practice from time immemorial down to 1866—the year the volume first appeared in London. J. H. E.

*A Treatise on Diseases of the Nervous System.* By WILLIAM A. HAMMOND, M.D., Professor of Diseases of the Mind and Nervous System, and Clinical Medicine, and of Materia Medica and Therapeutics, in the Bellevue Hospital Medical College, etc., etc. With 48 illustrations. Third edition, with additions and corrections. New York: D. Appleton & Co. 1872.

*Lessons in Physical Diagnosis.* By ALFRED L. LOOMIS, M.D., Professor of the Institutes and Practice of Medicine, in the Medical Department of the University of New York, etc., etc. Third edition, revised and enlarged. New York: William Wood & Co. 1872.

PAMPHLETS.

*Transactions of the Georgia Medical Association at its Twenty-Third Annual Meeting*, held in Columbus, Georgia, on the 10th, 11th and 12th of April, 1872.

*Modern Medicine:* a Lecture delivered Oct. 7th, 1872, introductory to the Course, at the Jefferson Medical College. By J. M. DACOSTA, M.D., Professor of the Principles and Practice of Medicine. Philadelphia: J. B. Lippincott & Co.

*Transactions of the Indiana State Medical Society*, 1872. Twenty-Second Annual Session.

*Transactions of the Twenty-Second Anniversary Meeting of the Illinois State Medical Society*, held at Rock Island, May 21st and 22nd, 1872.

*Transactions of the Minnesota State Medical Society*, 1872.

*Remarks on Strictures of the Urethra of Extreme Calibre*, with cases and a description of New Instruments for their treatment. By F. N. OTIS, M.D., Clinical Professor of Venereal Diseases in the College of Physicians and Surgeons, New York, etc., etc. Reprinted from *New York Medical Journal*, February, 1872. New York: D. Appleton & Co.

*On the Physiology of Syphilitic Infection.* By FESSENDEN N. OTIS, M.D. Reprinted from *The Medical Gazette.* New York: F. Leypoldt. 1872.

*On the Physiology of Syphilitic Infection as applied to the Successive Manifestations of the Disease.* By FESSENDEN N. OTIS, M.D. Part Second. Reprinted from *New York Medical Journal*, July, 1872. New York: D. Appleton & Co.

*Medical and General Science as Vindicators of the Mosaic Record, and as Repudiators of the Modern Doctrines of Development and Selection.* By E. S. GAILLARD, M.D., Professor of the Principles and Practice of Medicine in the Louisville Medical College, etc. (Reprint from October No. *Richmond and Louisville Medical Journal.*)

*What Physiological Value has Phosphorus as an Organismal Element?* An Essay to which was Awarded the Prize of the American Medical Association for the year 1872. By SAMUEL R. PERCY, M.D., Professor of Materia Medica in the New York Medical College, etc., etc., etc.

*An Illustrated Catalogue* of the Medical and Scientific Publications of William Wood & Co., New York, for 1872.

*Aiken (South Carolina) and its Climate.* By AMORY COFFIN, M.D., and W. H. GEDDINGS, M.D. 1872.

*School of Mines, Columbia College,* 1871-72. Catalogue of Students, etc.

*Second Annual Commencement* and Catalogue of the Thompson Free Medical College of New York, for Women. 1872.

*Proceedings of the Pathological Society of Philadelphia.* Vol. III. Philadelphia: 1871.

*Physicians' Visiting List for* 1873. Philadelphia: Lindsay & Blakiston.

---

**Treatment of Asthma.**

George Goskoin, surgeon to British Hospital for Diseases of the Skin, has had success in treating this disease by rubbing briskly into the chest, for the space of an hour, a chloroform liniment. The counter-irritation produced by the liniment was of benefit, but this benefit was increased by the jolting resulting from the rubbing. Anything that leads to the displacement of the air stagnant in the vesicles has proved able to relieve in many instances. It is advised that the friction be made with as much roughness as the case admits. Slight blows with the palm of the hand or the end of a towel on the ribs are quite allowable; and the friction should be extended to the front of the neck at the lower part, where the vagi enter the chest. The composition of the liniment need not trouble us, provided it be warm and work well.—*British Med. Jour.—The Detroit Review of Medicine.*

